# Potential Regulatory Role of MicroRNAs in the Development of Bovine Gastrointestinal Tract during Early Life

**DOI:** 10.1371/journal.pone.0092592

**Published:** 2014-03-28

**Authors:** Guanxiang Liang, Nilusha Malmuthuge, Thomas B. McFadden, Hua Bao, Philip J. Griebel, Paul Stothard, Le Luo Guan

**Affiliations:** 1 Department of Agricultural, Food and Nutritional Science, University of Alberta, Edmonton, Alberta, Canada; 2 Division of Animal Sciences, University of Missouri, Colombia, Missouri, United States of America; 3 Vaccine and Infectious Disease Organization, University of Saskatchewan, Saskatoon, Saskatchewan, Canada; 4 School of Public Health, University of Saskatchewan, Saskatoon, Saskatchewan, Canada; University of Houston, USA, United States of America

## Abstract

This study aimed to investigate the potential regulatory role of miRNAs in the development of gastrointestinal tract (GIT) during the early life of dairy calves. Rumen and small intestinal (mid-jejunum and ileum) tissue samples were collected from newborn (30 min after birth; n = 3), 7-day-old (n = 6), 21-day-old (n = 6), and 42-day-old (n = 6) dairy calves. The miRNA profiling was performed using Illumina RNA-sequencing and the temporal and regional differentially expressed miRNAs were further validated using qRT-PCR. Analysis of 16S rRNA gene copy numbers was used to quantify total bacteria, *Bifidobacterium* and *Lactobacillus* species. The expression of miR-143 was abundant in all three gut regions, at all time points and it targets genes involved primarily in the proliferation of connective tissue cells and muscle cells, suggesting a role in regulating rapid tissue development during the early life of calves. The expression of miR-146, miR-191, miR-33, miR-7, miR-99/100, miR-486, miR-145, miR-196 and miR-211 displayed significant temporal differences (FDR <0.05), while miR-192/215, miR-194, miR-196, miR-205 and miR-31 revealed significant regional differences (FDR <0.05). The expression levels of miR-15/16, miR-29 and miR-196 were positively correlated with the copy numbers of 16S rRNA gene of *Bifidobacterium* or *Lactobacillus* species or both (*P*<0.05). Functional analysis using Ingenuity Pathway Analysis identified the above mentioned differentially expressed miRNAs as potential regulators of gut tissue cell proliferation and differentiation. The bacterial density-associated miRNAs were identified as modulators of the development of lymphoid tissues (miR-196), maturation of dendritic cells (miR-29) and development of immune cells (miR-15/16). The present study revealed temporal and regional changes in miRNA expression and a correlation between miRNA expression and microbial population in the GIT during the early life, which provides further evidence for another mechanism by which host-microbial interactions play a role in regulating gut development.

## Introduction

The gastrointestinal tract (GIT) of mammals undergoes microbial colonization immediately after birth [1] and thereafter is continuously exposed to commensal microbiota, pathogens, and dietary antigens. There is increasing evidence that the gut microbiota plays a vital role in regulating host immune functions [2]. The host mucosal immune system prevents bacterial invasion and shapes the gut microbiota, while the gut microbiota influences immune system development [2]. Recent studies using mouse models reveal the molecular basis for the complex and dynamic interactions between commensal bacteria and the mucosal immune system [3], [4]. Toll-like receptors (TLRs) signaling pathway is one of the innate immune responses that recognizes microbial-associated molecular patterns (MAMPs) and activates downstream intracellular signals [5]. It also maintains host-microbial homeostasis by modulating proliferation of epithelial cells and/or secretion of antimicrobial proteins [6], [7]. Moreover, gut microbial colonization also has profound effects on the differentiation of T cells. For example, bacterial colonization of germ-free mice enhanced the differentiation of T helper 17 cells (Th17) and regulatory T cells (Tregs) [8], [9]. However, the molecular mechanisms involved in regulating these interactions remain largely undefined.

MicroRNAs (miRNAs) are small (∼22 nucleotides) endogenous RNAs that regulate gene expression by targeting the 3′ untranslated region (3′UTR) [10] and/or coding region [11] of mRNAs. They have been reported to be involved in the regulation of many biological processes including embryo development, cell differentiation, apoptosis and metabolism [12]. The expression of miRNAs is ubiquitous among mammalian cells; however, their expression patterns and regulatory roles can be tissue and/or species specific [13]–[15]. miRNAs regulate genes as part of the complex regulatory networks in the immune system, by playing pivotal roles in the regulation of both immune cell development and effector functions [16]. For example, miR-196 induces the cleavage of *homeobox* mRNA in hematopoietic stem cells (HSCs) and modulates HSC homeostasis [17], [18]. Besides, they regulate gut homeostasis and mucosal immunity. MiR-375 regulates T cell subset amplification in the murine colon [19]. The expression of miR-155, miR-146 and miR-21 can be induced by TLR activation and target the 3′UTR of mRNAs encoding TLR signalling pathway molecules, such as IL-1R-associated kinase 1 and TNFR-associated factor 6 [20]. In addition, recent studies reported that miRNAs may be involved in host-microbial interactions. The up-regulation of miR-27b through TLR4/NF-κB signalling following a gut protozoan infection was observed and it suppressed expression of KH-type splicing regulatory protein that is vital for antimicrobial activity [21]. MiR-665, reported to be up-regulated during microbial colonization of germ-free mice, regulates mRNA of ATP-binding cassette, sub-family c, member 3, a protein that mediates the metabolism of xenobiotics and endogenous toxins in intestine [22]. However, the roles of miRNA in host-microbial interactions during early life have not been reported. We hypothesized that miRNAs coordinate immune system development of the host in response to microbial colonization in the GIT during early life of calves.

This study therefore, 1) analyzed temporal and regional miRNA profiles throughout the GIT during the first 6 weeks after birth; 2) explored the potential roles of miRNAs in mucosal immune system development by identifying predicted target genes, and lastly; 3) investigated whether miRNAs expression patterns are temporally and regionally associated with changes in the GIT microbial population in the dairy calves.

## Materials and Methods

### Animal Study and Sample collection

Small intestine (mid-jejunum and ileum) and rumen samples were collected from newborn (D0, n = 3), 7-day-old (D7; n = 6), 21-day-old (D21; n = 6) and 42-day-old (D42; n = 6) male Holstein calves at Dairy Research and Technology Center (DRTC), University of Alberta. All experimental protocols were reviewed and approved by the Livestock Animal Care committee of the University of Alberta (protocol no. LS150) and all procedures were conducted following the guidelines of Canadian Council on Animal Care. D0 samples were collected from newborn animals, within 30 min after delivery without feeding colostrum. The D7 calves were fed with only whole milk (4 l/day), while D21 and D42 calves received whole milk with *ad libitum* access to calf starter (23% CP and 4% ether extract (EE) as the guaranteed minimum, 19.5% NDF, 27.1% starch - Wetaskiwin Co-Op Country Junction, Wetaskiwin, AB, Canada). All calves were euthanized using captive bolt gun, and samples were collected within 30 min after euthanization. Ileum was defined as 30 cm proximal to ileo-cecal junction and 10 cm in the middle of the 30 cm segment was collected. Mid-jejunum was defined as 1 m distal to the pyloric sphincter and 10 cm in the middle of the 1 m segment was collected. Rumen tissue samples were collected from the lateral wall (∼10 cm^2^). Tissue and digesta samples were collected from the same site separately after euthanization, snap-frozen in liquid nitrogen and stored in −80°C. Due to very limited volume of the contents in D0 samples, tissue and content were processed together.

### Nucleic acid isolation

Tissue samples were ground into fine powder while immersed in liquid nitrogen prior to nucleic acid extraction. Total RNA was extracted from 80 mg of tissue using mirVana miRNA Isolation Kit (Ambion, Carlsbad, CA) following the manufacturer's instructions. The quality and quantity of the RNA were determined using Agilent 2100 Bioanalyzer (Agilent Technologies, Santa Clara, CA) and Qubit 2.0 Fluorometer (Invitrogen, Carlsbad, CA), respectively. RNA samples with integrity number (RIN) higher than 7.0 were used for further analysis.

Total DNA was extracted from tissue samples (∼100 mg) using the bead beating method as described by Li et al [23]. Briefly, samples were subjected to physical disruption in a BioSpec Mini Beads beater 8 (BioSpec, Bartlesville, OK) at 5000 rpm for 3 min, followed by phenol:chloroform:isoamyl alcohol (25∶24∶1) extraction. DNA was then precipitated with cold ethanol and dissolved in nuclease free molecular grade water. The quantity and quality of DNA was measured using ND1000 spectrophotometer (NanoDrop Technologies, Wilmington, DE).

### MiRNA library construction and sequencing

Total RNA (1.0 μg each) from each sample was used to construct miRNA library with an unique index using the TruSeq Small RNA Sample Preparation kit (Illumina, San Diego, CA) according to the manufacturer's instruction. PCR amplification was performed for 11 cycles and libraries with unique indices were purified individually using gel purification. Quantitative real time PCR (qPCR) was performed for library quantification using the StepOnePlus Real-Time PCR System (Applied Biosystems, Carlsbad, CA) and KAPA SYBR Fast ABI Prism qPCR kit (Kapa Biosystems, Woburn, MA). The diluted libraries were loaded on cBot (Illumina) for cluster generation using the TruSeq SR Cluster kit v3 (Illumina). Sequencing was performed on the HiScan SQ system (Illumina) using the TruSeq SBS kit v3 (50 cycles, Illumina). Real-time analysis and base calling was performed using the HiSeq Control Software Version 1.4.8 (Illumina).

### Sequencing data analysis

Low-quality reads were removed from raw data using CASAVA 1.8 based on chastity, and the sequences with good quality were then subjected to 3′ adaptor sequence trimming. Then, the sequences of sizes ranging from 18 to 30 nt were mapped to the ncRNA sequences (Rfam) to remove non-miRNA sequences such as tRNA, snoRNA, rRNA, and other non-coding RNAs. The known and novel miRNAs were identified using miRDeep2 [24] based on a probabilistic model of miRNA biogenesis [25]. For the known miRNAs, the filtered sequences were aligned against the corresponding known miRNA precursor sequences (miRBase release version 19) by using the module of quantifier.pl in miRDeep2 with the default parameters to identify known miRNAs. The known miRNAs with total number of reads above 20 were defined as expressed known miRNAs. Novel miRNAs were detected using miRDeep2, with the miRDeep2 score cutoff of 5 and more than 20 mapped reads from all libraries. Each library was processed separately, and the results of each novel miRNA candidate were combined together according to genomic location. The novel miRNAs from different locations within the bovine genome that contain same sequence were reported as single novel miRNA candidate.

The conservation of known miRNAs was analyzed based on the TargetScan definitions for “highly conserved”, “conserved”, and “poorly conserved” [26]. A highly conserved miRNA means that it is conserved across most vertebrates; a conserved miRNA is conserved across most mammals, but usually not beyond placental mammals; a poorly conserved miRNA is not present in above two groups. In this study, bovine specific miRNAs were defined by using two conditions: (1) miRNAs belong to poorly conserved group; (2) miRNAs with seed region sequences only reported in cattle.

Temporal and regional effects on the miRNA expression were investigated by characterizing differentially expressed (DE) miRNAs using bioinformatics tool edgeR [27], which utilizes a negative binomial distribution to model sequencing data. The expression of miRNAs in each library was normalized to counts per million reads (CPM) by the following method: CPM  =  (miRNA reads number/total reads number per library) ×1,000,000. For each comparison, miRNAs with CPM >5 in at least 50% of the samples were subjected to DE analysis. Fold changes were defined as ratios of arithmetic means of CPM within each comparison group. The significant DE miRNAs were determined by FDR <0.05 based on Benjamini and Hochberg multiple testing correction [28] as well as fold change >1.5.

### Estimation of the total bacterial and two beneficial bacterial populations using qPCR

qPCR was performed using SYBR Green chemistry (Fast SYBR® Green Master Mix; Applied Biosystems) to estimate the tissue-attached total bacterial population using the copy number of 16S rRNA gene. Total bacterial population was estimated using U2 primers (U2F, 5′-ACTCCTACGGGAGGCAG-3′; U2R, 5′-GACTACCAGGGTATCTAATCC-3′) [29] with StepOnePlus Real-Time PCR System (Applied Biosystems). The standard curve was constructed using plasmid DNA containing 16S rRNA gene of *Butyrivibrio hungatei* with a serial dilution of initial concentration of 8.5×10^10^ mol/μl. The populations of *Lactobacillus* (Lac) and *Bifidobacterium* (Bif) spp. were estimated using Lac primers and Bif primers (Lac1: 5′-AGCAGTAGGGAATCTTCCA-3′ and Lac2: 5′-ATTTCACCGCTACACATG-3′ [30]; Bif1: 5′-CGTCAAGCTGCTAGGACGC-3′ and Bif2: 5′-TACACCGGAATAGCTCCTGG-3′) (designed in this study). The standard curves were constructed using serial dilutions of genomic DNA from *Lactobacillus acidophilus* and *Bifidobacterium longum* with serial dilutions of the initial concentration of 5.8×10^8^ and 2.05×10^7^ mol/μl^−1^, respectively. The copy number of 16S rRNA gene per gram sample for each targeted bacterial population was calculated using the method reported in a previous study [31]: (QM × C × DV)/(S × W), where QM was the quantitative mean of the copy number, C was the DNA concentration of each sample (ng μL^−1^), DV was the dilution volume of extracted DNA (μL), S was the DNA amount subjected to analysis (ng), and W was the sample weight subjected to DNA extraction (g).

### Correlation between miRNAs expression and bacterial density

The possible relationships between miRNA expression and microbial colonization in the GIT were explored using Pearson's correlation in R software and COR function. Normalized miRNAs expression (CPM) and log_10_ copy number of 16S RNA genes of total bacteria, *Lactobacillus* and *Bifidobacterium* spp. were used to define the correlations in each gut region under each time point. In the rumen the correlations were done only between total bacterial density and miRNAs expression. The significant correlations were declared at *P*<0.05, r > 0.8 or r < −0.8.

### Experimental validation of miRNA expression using stem-loop qRT-PCR

The expression of regional and temporal DE miRNAs that identified by miRNA-seq was validated by stem-loop qRT-PCR using TAQMAN miRNA assays following the manufacturer's recommendation (Applied Biosystems). Briefly, cDNAs were reverse transcribed from 10 ng of total RNA using 5 X specific miRNA RT primer and then same amount of cDNAs were amplified using a 20 X TAQMAN miRNA assay. Fluorescence signal was detected with StepOnePlus Real-Time PCR System (Applied Biosystems). In this study, U6 snRNA was used as an internal control to calculate the relative expression of selected miRNAs following the formula: ΔCt_target miRNA_  = Ct_target miRNA_–Ct_U6_. Lower ΔCt represent higher miRNA abundance level and higher ΔCt represent lower miRNA abundance level. T-test was used to compare difference between each comparison group (regional DE miRNA: rumen *vs* mid-jejunum, rumen *vs* ileum and mid-jejunum *vs* ileum; temporal DE miRNA: D7 *vs* D0, D21 *vs* D7, D42 *vs* D21). The statistical significances were declared at *P*<0.05 and analyses were performed in R using t.test function.

### MiRNA targets prediction and functional analysis

Target genes for selected miRNAs were predicted by using TargetScan Release 6.0 (http://www.targetscan.org/) [32] and miRanda (http://www.microrna.org/microrna). The target genes that were predicted by both TargetScan (default parameters) and miRanda (Total score> = 145, Total energy< = −10) for each miRNA were further analyzed through IPA (Ingenuity Systems, www.ingenuity.com). The multiple testing corrected *P* value calculated by Benjamini-Hochberg method (FDR) [28] was used to determine the significance of the predicted function in IPAs. A threshold of FDR <0.05 and molecule number >1 were applied to enrich significant biological functions of each miRNA.

### Data Submission

All the sequencing data were deposited in the publicly available NCBI's Gene Expression Omnibus Database (http://www.ncbi.nlm.nih.gov/geo/). The data are accessible through GEO Series accession number GSE52193 (http://www.ncbi.nlm.nih.gov/geo/query/acc.cgi?acc=GSE52193).

## Results

### Profiling of miRNAs in rumen and small intestine of dairy calves

MiRNA expression was studied by sequencing 63 small RNA libraries prepared from ruminal, mid-jejunal and ileal tissues collected from newborn calves within 30 min after delivery (D0; n = 3), 7-day-old calves fed milk (D7; n = 6), 21-day-old (D21; n = 6) and 42-day-old calves fed with milk supplemented with calf starter. A total of 97 million high-quality small RNA reads were obtained, with a median of 1.4 million reads per library (ranged from 0.6 million to 3.8 million). The majority of sequences were 21nt and 22nt in size and accounted for 62.7% of total reads (Figure S1).

**Figure 1:**
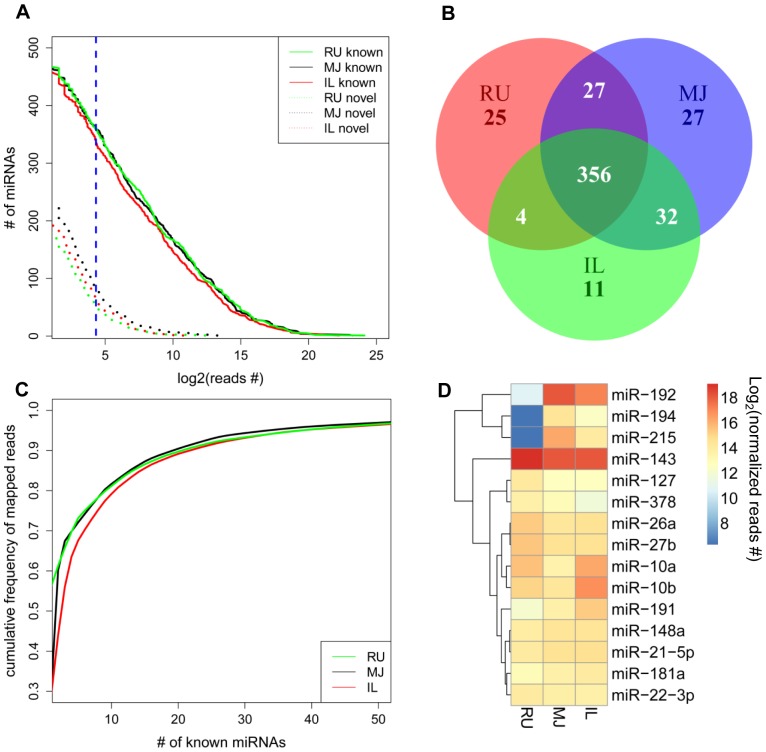
Overview of miRNAs detected in the GIT of dairy calves during early life using miRNA sequencing. (A). Numbers of known (solid line) and novel miRNAs (dotted line) identified (total reads number for each tissue >20, blue line). (B). Comparison of the number of known miRNAs detected in rumen (RU), mid-jejunum (MJ), and ileum (IL). (C). Cumulative frequency of known miRNAs detected in rumen (RU), mid-jejunum (MJ), and ileum (IL). (D). Comparative expression of top 10 highly expressed miRNAs in rumen (RU), mid-jejunum (MJ), and ileum (IL) using Heatmap.2 function in R package. Colors represent different normalised sequencing reads number as indicated by the color bar.

**Figure 2:**
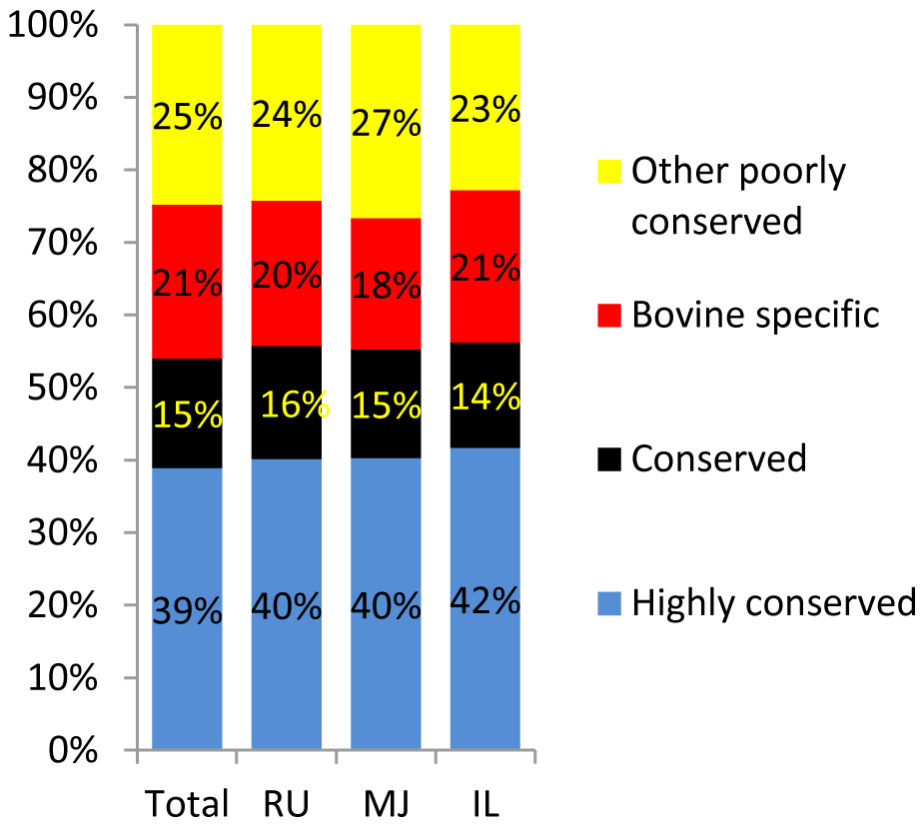
Conservation analysis of known miRNAs detected in calf GIT. Proportion of different miRNAs conservation categories in total identified known miRNAs (Total), in rumen (RU), in mid-jejunum (MJ), and in ileum (IL). Highly conserved (blue bar): conserved across most vertebrates; Conserved (black bar): conserved across most mammals, but usually not beyond placental mammals; Poorly conserved: miRNAs that do not belong to the above two groups, including bovine specific (red bar, belong to poorly conserved group, and seed region sequence only reported in cattle) and other poorly conserved miRNAs (yellow bar).

In total, 77 million out of 97 million reads were mapped to the known miRNA database (miRBase release version 19 [33]) and 0.9 million reads were identified as novel miRNAs, resulting in the identification of 383 known miRNAs and 169 putative novel miRNAs from all libraries (Table S1). Furthermore, 359 known and 53 novel miRNAs in rumen, 360 known and 82 novel miRNAs in mid-jejunum, and 338 known and 65 novel miRNAs in ileum (Figure 1A) were identified from all the calves. Among all identified miRNAs, 356 known miRNAs were detected in all three GIT regions (Figure 1B). The top 10 expressed miRNAs represented more than 80% of the total mapped reads in each tissue (Figure 1C & 1D). The predominant miRNAs in the mid-jejunum were miR-192 (33%), -143 (28%), and -215 (7%), and in ileum were miR-143 (30%), -192 (15%), -10a (12%) and -10b (8%). Only one predominant miRNA, miR-143, which accounted for 60% of total mapped reads, was detected in the rumen (Figure 1D). Among the total known miRNAs (383) identified in the calf GIT, 39% (141) and 15% (58) of them belonged to highly conserved and conserved, respectively, while 46% of them (176) belonged to poorly conserved groups (Figure 2). Based on the analysis for each individual gut region, the present study also detected 81 bovine specific miRNAs (Figure 2).

### Identification of temporally differentially expressed miRNAs

**Figure 3:**
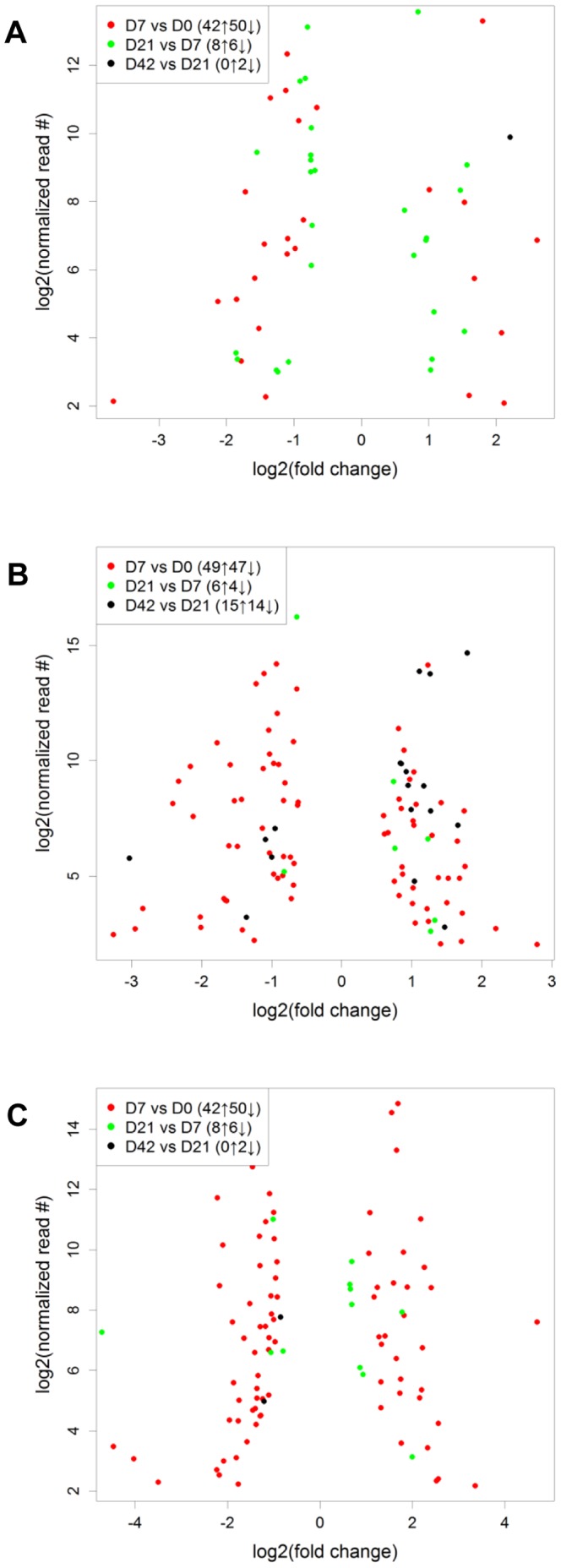
Detected temporally DE miRNAs based on comparisons D7 *vs.* D0 (red), D21 *vs.* D7 (green), and D42 *vs.* D21 (black). The X and Y axes show log2 (fold change) and log2 (normalised reads number) of each DE miRNA, respectively. (A). DE miRNAs detected in rumen (RU) tissue. (B). DE miRNAs identified in tissue collected from mid-jejunum (MJ). (C). DE miRNAs detected in tissue collected from from ileum (IL). “↑”: the number of up-regulated miRNAs; “↓”: the number of down-regulated miRNA.

Temporal effect on miRNA expression was examined independently for each tissue by performing the following comparisons: D7 *vs* D0, D21 *vs* D7, and D42 *vs* D21 and the differentially expressed (DE) miRNAs are shown in Table S2. Among all comparisons, more DE miRNAs were detected when comparing D7 *vs* D0 in the mid-jejunum (Figure 3B) and ileum (Figure 3C) as well as from D21 *vs* D7 in the rumen (Figure 3A). The temporal effect was confounded by the effect of changing diet. To minimize the dietary effect, we selected DE miRNAs present in each tissue in at least two out of three of the temporal comparisons for further functional analysis. MiRNA candidates were defined at family level, since miRNAs belonging to the same family are considered to perform similar functions based on the same seed sequence [32]. A total of 8 DE miRNAs belonging to 8 miRNA families for rumen, 20 DE miRNAs belong to 16 miRNA families for mid-jejunum, and 6 DE miRNAs belonging to 6 miRNA families for ileum were selected for further functional analysis (Table S4).

### Identification of regionally differentially expressed miRNAs

**Figure 4:**
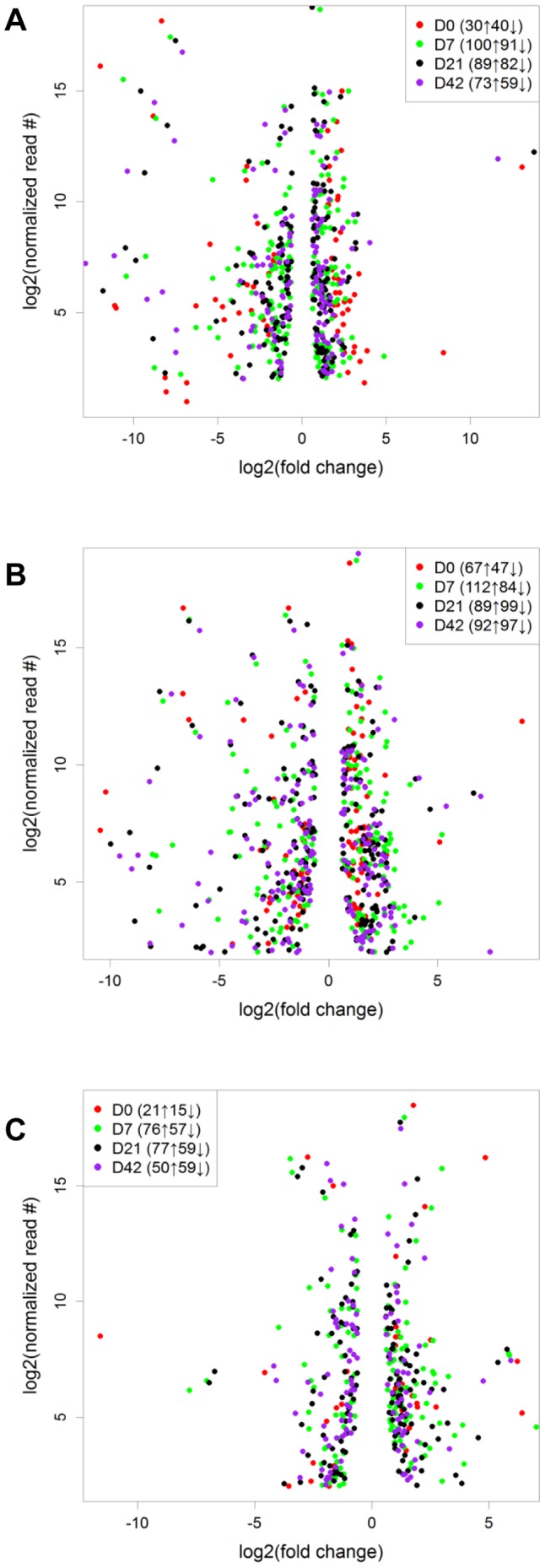
Detected regionally DE miRNA based on comparisons between different tissues on D0 (red), D7 (green), D21 (black), and D42 (purple). (A). Detected DE miRNAs when comparing rumen (RU) *vs.* mid-jejunum (MJ). (B). Detected DE miRNAs when comparing rumen (RU) *vs.* ileum (IL). (C). Detected DE miRNAs when comparing mid-jejunum (MJ) *vs.* ileum (IL). “↑”: the number of up-regulated miRNAs; “↓”: the number of down-regulated miRNA.

**Figure 5:**
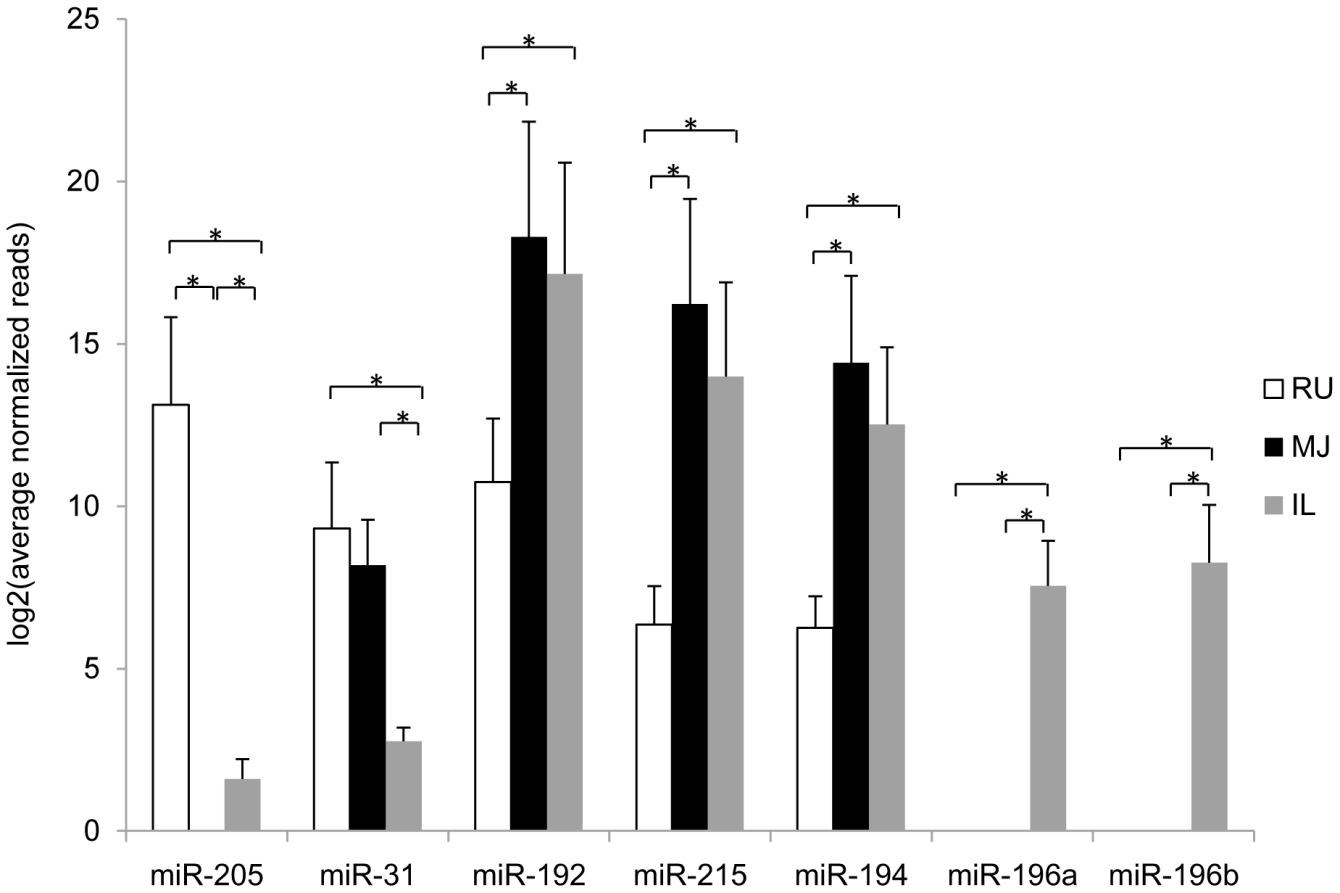
Expression levels of the most significant regionally DE miRNAs detected by miRNA sequencing in rumen (RU, open bar), mid-jejunum (MJ, solid black bar) and ileum (IL, solid grey bar). “*” represents miRNA differentially expressed between two tissues (FDR <0.05, >1.5 fold). No bar is shown when miRNA expression was not detected.

MiRNA expression was further compared among GIT regions at each developmental stage. As shown in Figure 4, more DE miRNAs were identified in rumen *vs* mid-jejunum (Figure 4A) and rumen *vs* ileum (Figure 4B) compared to ileum *vs* mid-jejunum (Figure 4C) at all the time points. First, DE miRNAs that were common to all three developmental stages within a specific GIT region were identified: 42 families (45 DE miRNAs) were from rumen *vs* mid-jejunum, 80 families (87 DE miRNAs) were from rumen *vs* ileum, and 15 families (18 DE miRNAs) were from ileum *vs* mid-jejunum (Table S3). Then, 5 families (7 DE miRNAs) that had the most significant (FDR <0.05) DE patterns were subjected to functional analysis (Table S4). Among these 5 miRNA families, miR-192/215 and miR-194 were highly expressed in the small intestine (mid-jejunum and ileum), while miR-205 was highly expressed in the rumen but not detected in mid-jejunum (Figure 5). The miR-196 family was only detected in ileum, while miR-31 had lower expression in the ileum compared to the mid-jejunum and rumen (Figure 5).

### Association between miRNA expression and gut microbial population

**Figure 6:**
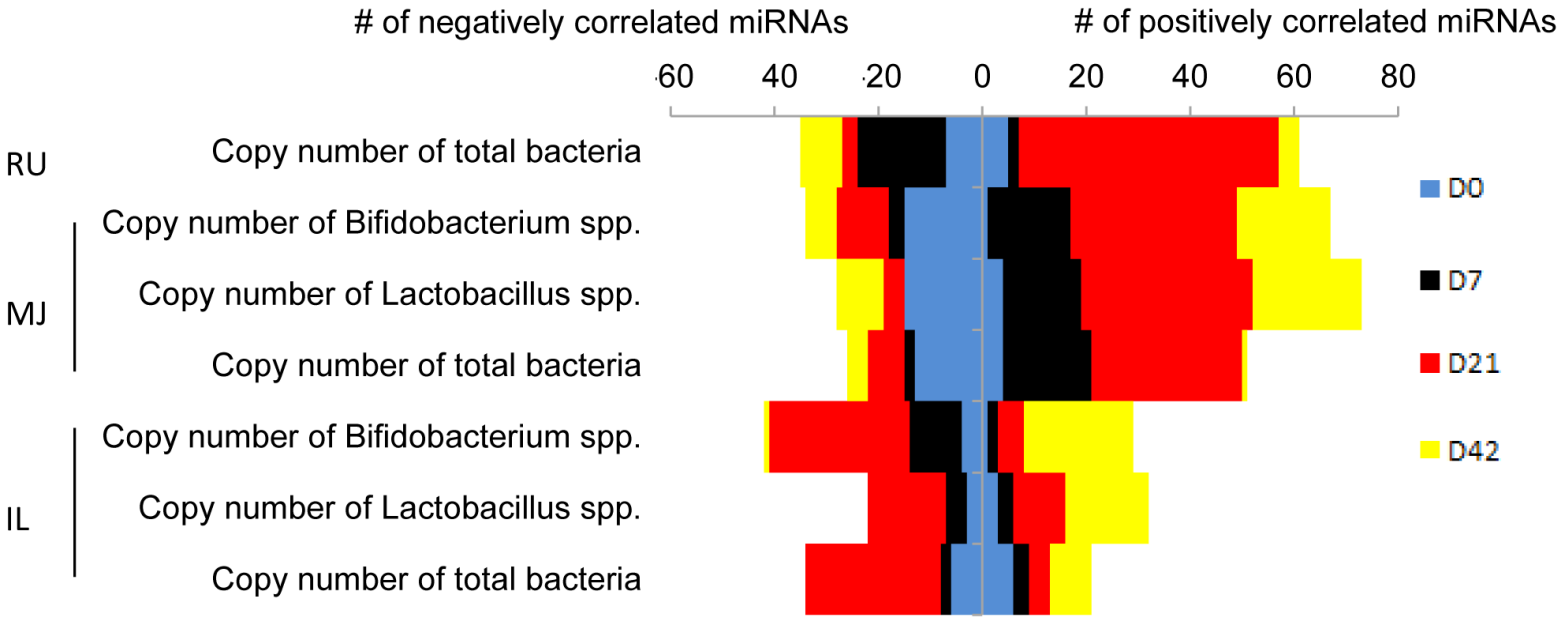
Numbers of miRNAs with significant correlations between their expression and the copy number of microbial 16S rRNA gene. The correlation analysis was performed between normalized miRNAs expression and log copy number of total bacteria, *Lactobacillus* and *Bifidobacterium* spp. in each location (RU: rumen; MJ: mid-jejunum; IL: ileum) and at each time point (D0: blue bar; D7: black bar; D21: red bar; D42: yellow bar). The horizontal axis shows the number of miRNAs correlated with microbial density. “# of negatively correlated miRNAs” means the number of miRNAs with a negative correlation (P<0.05, r<−0.8) with microbial density. “# of positively correlated miRNAs” means the number of miRNAs with a positive correlation (*P*<0.05, r>0.8) with microbial density.

The expression of miRNAs was strongly correlated with the density of total bacteria, *Bifidobacterium* and *Lactobacillus* spp. (Figure 6) throughout the GIT at all stages. At D21, there were more miRNAs significantly correlated with bacterial density than other groups (Figure 6). Four miRNA families associated with bacterial density were selected for further functional analysis (Table S4): the miR-129 family, which revealed positive correlation with total bacterial density (r = 0.90, *P* = 0.01) in the rumen on D7 and D21; three miRNA families from ileum including the miR-15/16 family (negatively correlated with the density of *Bifidobacterium* spp. on D21; r = −0.87, *P* = 0.01), the miR-196 family (positively correlated with the population of both *Bifidobacterium* and *Lactobacillus* spp. on D21, r = 0.83, *P* = 0.02), and the miR-29 family (positively correlated with the population of both *Bifidobacterium* and *Lactobacillus* spp. on D42; r = 0.95, *P*<0.01).

### Experimental validation of miRNA expression using stem-loop qRT-PCR

**Figure 7:**
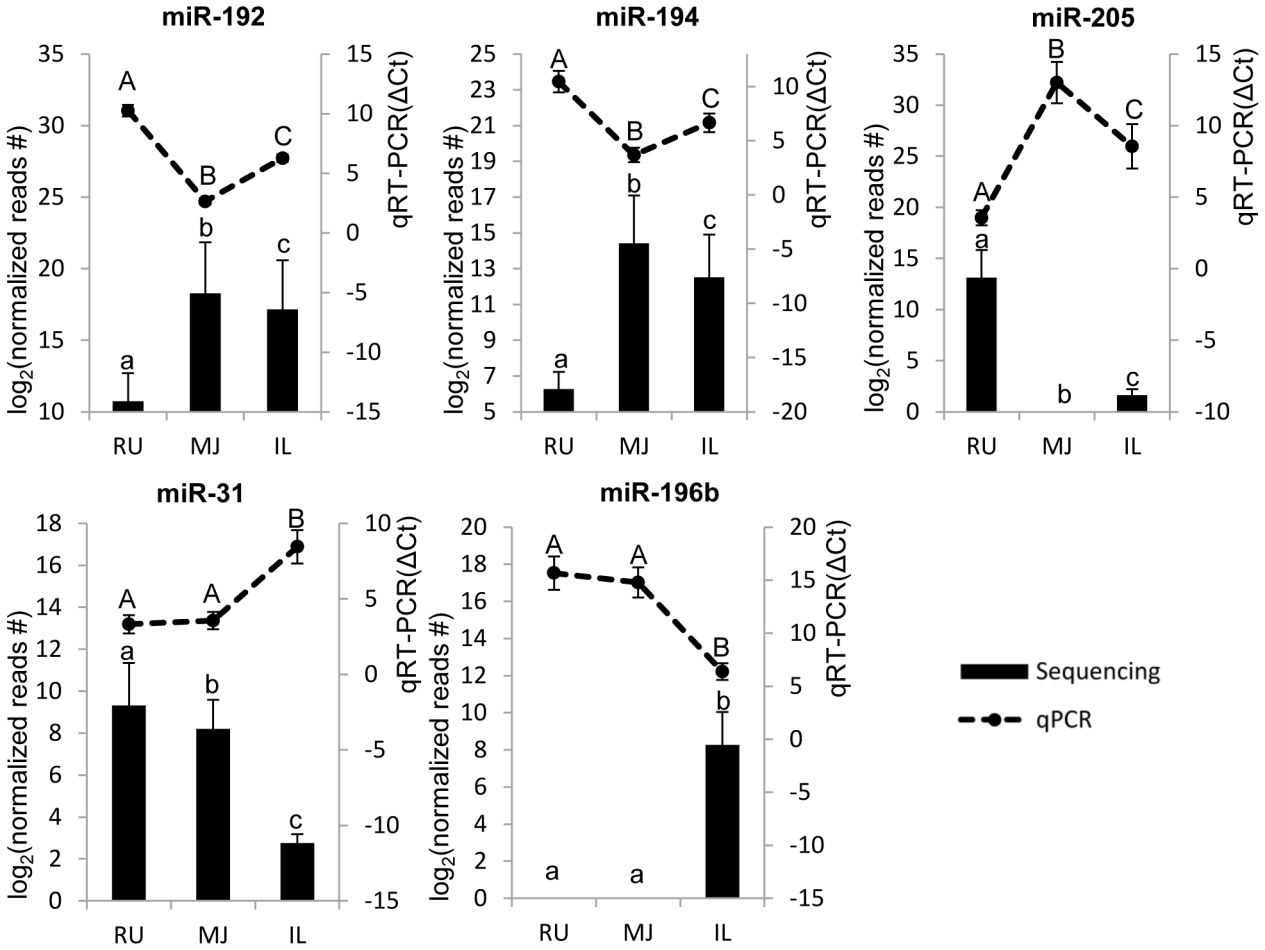
Expression of regional DE miRNAs detected by qRT-PCR and miRNA-seq. MiRNA expressions from qRT-PCR are shown by line graphs on the top and values are shown on the right Y-axis as relative expression (ΔCt). Lower ΔCt values represent higher miRNA expression levels and *vice versa*. MiRNA expressions from miRNA-seq are shown by bar graphs on the bottom and values are shown on the left Y-axis as log_2_ (normalised reads number). A, B, C - indicate the significant difference in the relative expression of miRNAs detected via qRT-PCR at *P*<0.05; a, b, c - indicate significant difference in the expression of miRNAs detected from miRNA-seq at FDR <0.05. Data are presented as Mean±Standard deviation. RU, MJ and IL represent rumen, mid-jejunum, and ileum, respectively.

**Figure 8:**
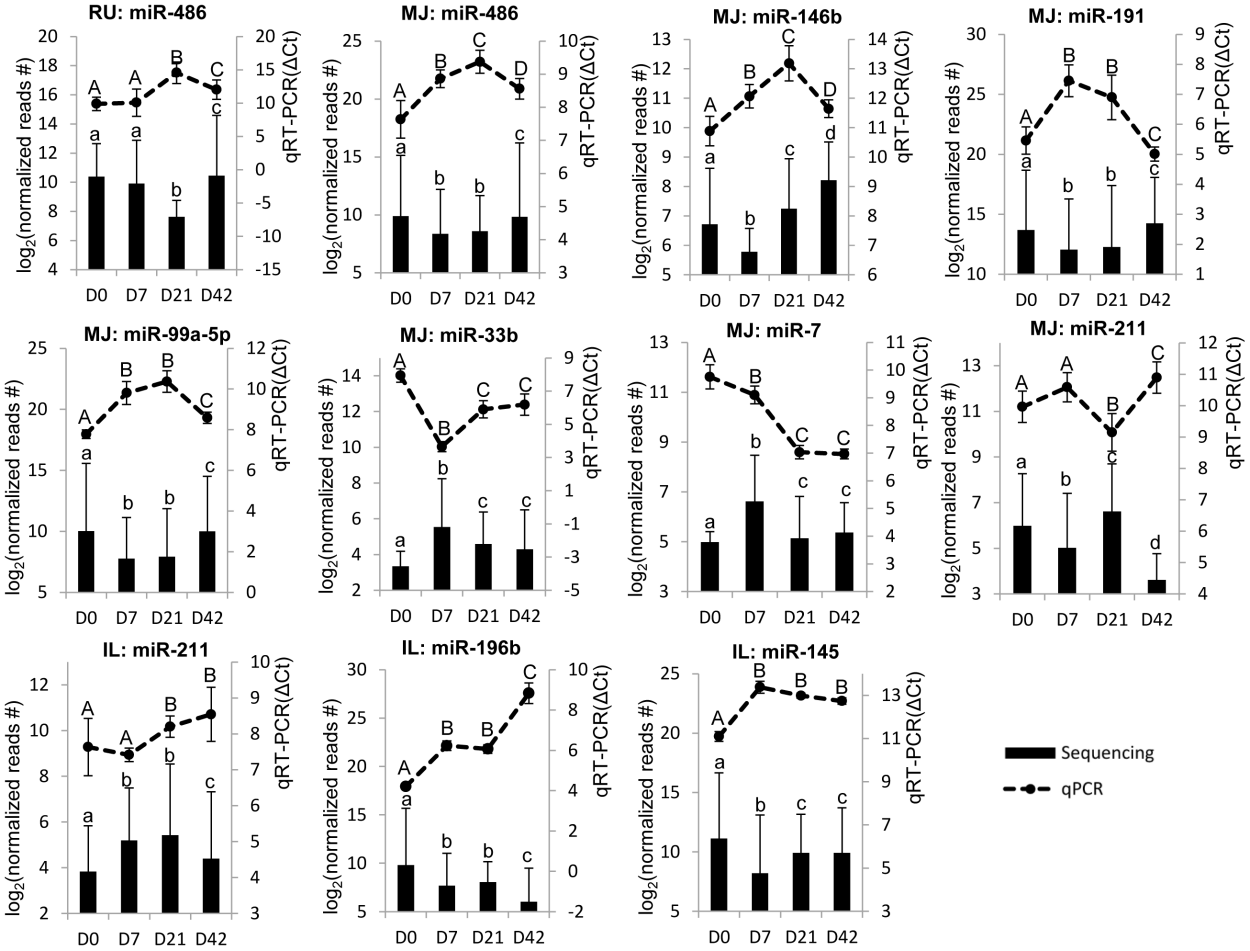
Expression of temporal DE miRNAs detected by qRT-PCR and miRNA-seq. MiRNA expressions from qRT-PCR are shown by line graphs on the top and values are shown on the right Y-axis as relative expression (ΔCt). Lower ΔCt values represent higher miRNA expression levels and *vice versa*. MiRNA expressions from miRNA-seq are shown by bar graphs on the bottom and values are shown on the left Y-axis as log_2_ (normalised reads number). A, B, C, D - indicate significant expression difference between pairs at *P*<0.05 (D7 *vs* D0, D21 *vs* D7, D42 *vs* D21) detected via qRT-PCR. a, b, c, d - indicate significant expression difference between pairs at FDR <0.05 (D7 *vs* D0, D21 *vs* D7, D42 *vs* D21) detected by miRNA-seq. Data are presented as Mean±Standard deviation.

A total of 13 regional and temporal DE miRNAs (regional DE miRNAs: miR-192, miR-194, miR-205, miR-31, and miR-196; temporal DE miRNAs: miR-146b, miR-191, miR-99a, miR-145, miR-211, miR-486, miR-33, miR-7, and miR-196b) that identified from miRNA-seq were selected for validation using stem-loop qRT-PCR. The expressions of miR-192 and miR-194 were notably higher in the small intestine than that in the rumen. MiR-205 was highly expressed in the rumen, while miR-31 was highly expressed in the rumen and mid-jejunum not in the ileum. The expression of miR-196b cannot be detected in rumen and mid-jejunum by both miRNA-seq and qPCR. Generally, the regional DE miRNAs showed similar trend in the comparison between miRNA-seq and qPCR (Figure 7). Most of temporal DE miRNAs showed the expression in agreement between miRNA-seq and qRT-PCR; however, three of them (miR-486, miR-146b, miR-7, and miR-211) revealed opposite trends between miRNA-seq and qRT-PCR in some comparison groups (Figure 8). For example, in mid-jejunum, miR-486, miR-146b and miR-7 had divergent regulation from D7 to D21; in ileum, miR-211 revealed significant down-regulation from D7 to D21 by qRT-PCR, but there was no significant difference identified by sequencing.

### Functional predictions of differentially expressed miRNAs

Based on predicted targets of miRNAs using TargetScan and miRanda as well as functional analysis using IPA, the significantly enriched (FDR <0.05, molecule number >1) function categories of a total of 33 selected miRNA families were identified (Table S4). Among these, a total of 9 function categories, include cellular development, cellular growth and proliferation, connective tissue development, digestive system development, hematological system development and function, immune response, inflammatory response, immune cell trafficking, and lymphoid tissue structure and development, were defined to be related to GIT gut development and immune system development. Fifteen out of the 33 selected miRNA families were predicted to have functions significantly associated with the GIT development or immune functions (Table 1). The function of the predominant miR-143 was predicted to be the differentiation of connective tissue cells, which was related to GIT development (Table 1). Temporally DE miRNAs in small intestine (miR-146, miR-191, miR-33, miR-7, miR-99/100, miR-486, miR-145, and miR-211) were predicted to be related to gut epithelial cells development, immune cells development, inflammatory response, and other functions (Table 1). However, most of the temporally DE miRNAs in the rumen did not show any significant predicted functions. Two regionally DE miRNA families (miR-194 and miR-192/215) revealed significantly enriched functions related to GIT development and immune system, such as formation of mast cells and differentiation of leukocytes (Table 1). In addition, miR-129, miR15/16, and miR-29 that correlate with bacterial populations may also regulate gut development, immune and digestive functions (Table 1). MiR-129 was predicted to play a role in rumen development in response to bacterial colonization. In addition, the temporally DE miRNA-196 was only detected in ileum (Table 1), and was significantly correlated with bacterial density (data not shown). This miRNA has known functions related to epithelium differentiation, lymphoid tissue development, and inflammatory response (Table 1).

**Table 1:** Enriched gut development-related and immune function-related functions of selected miRNAs.

miRNA families	miRNA Category *	Function Category	Functions Annotation	FDR	Molecules #
miR-143	A	Cellular Development	Differentiation of Connective Tissue Cells	4.98E-02	3
miR-146	B	Cellular Development	proliferation of enterocytes	3.67E-02	2
		Cellular Growth and Proliferation	proliferation of cells	4.47E-02	8
		Digestive System Development and Function	quantity of Paneth cells	3.67E-02	2
		Inflammatory Response	TH1 immune response	2.66E-02	3
		Inflammatory Response	phagocytosis by dendritic cells	3.03E-02	2
miR-191	B	Digestive System Development and Function	morphology of intestinal villus	1.44E-02	2
miR-33	B	Cellular Development	differentiation of cells	4.60E-02	5
		Hematological System Development and Function	accumulation of neutrophils	3.43E-02	2
		Inflammatory Response	phagocytosis of cells	2.54E-02	4
miR-7	B	Cellular Development	Differentiation of Muscle Cells	4.99E-02	2
miR-99/100	B	Cellular Development	Differentiation of Cells	3.35E-02	6
		Hematological System Development and Function	Differentiation of T Lymphocytes	4.66E-02	2
miR-486	C	Connective Tissue Development and Function	Proliferation of Fibroblast Cell Lines	4.53E-02	4
miR-145	D	Hematological System Development and Function	Differentiation of Leukocytes	4.92E-02	20
miR-211	E	Hematological System Development and Function	Differentiation of T Lymphocytes	3.64E-03	17
		Inflammatory Response	Il-6 Signaling	2.45E-02	3
		Inflammatory Response	Il-17 Signaling	1.58E-02	2
miR-194	F	Hematological System Development and Function	Colony Formation of Mast Cells	2.41E-02	2
miR-192/215	G	Hematological System Development and Function	Differentiation of Leukocytes	4.71E-02	8
		Hematological System Development and Function	Expansion of Leukocytes	4.62E-02	5
		Lymphoid Tissue Structure and Development	Proliferation of Lymphatic System Cells	4.42E-02	6
		Hematological System Development and Function	Expansion of Leukocytes	4.62E-02	5
		Lymphoid Tissue Structure and Development	Organogenesis of Lymphatic System Component	4.62E-02	2
miR-129	H	Digestive System Development and Function	Development of Digestive System	1.21E-02	2
miR-15/16	I	Cellular Growth and Proliferation	Proliferation of Cells	2.45E-02	16
		Hematological System Development and Function	Development of Leukocytes	4.39E-02	12
		Hematological System Development and Function	Development of Lymphocytes	2.72E-02	10
		Hematological System Development and Function	T Cell Development	3.71E-02	11
		Cellular Development	Differentiation of Cells	2.45E-02	6
		Hematological System Development and Function	Development of B Lymphocytes	1.58E-02	7
miR-29	J	Cellular Development	Differentiation of Epithelial Cells	2.29E-02	7
		Connective Tissue Development and Function	Proliferation of Fibroblasts	2.59E-02	7
		Connective Tissue Development and Function	Quantity of Connective Tissue Cells	2.29E-02	6
		Hematological System Development and Function	Maturation of Dendritic Cells	4.75E-02	2
miR-196	K	Cellular Growth and Proliferation	Proliferation of Epithelial Cells	3.84E-02	4
		Lymphoid Tissue Structure and Development	Proliferation of Lymphatic Endothelial Cells	4.63E-02	2
		Inflammatory Response	Morphology of Phagocytes	4.86E-02	2

*miRNA categories were represented by different letters: A: Predominant miRNA expressed in GIT; B: Temporal DE miRNA in MJ; C: Temporal DE miRNA in RU and MJ; D: Temporal DE miRNA in IL; E: Temporal DE miRNA in MJ and IL; F: Regional DE miRNA (RU vs IL; RU vs MJ); G: Temporal DE miRNA in MJ; Regional DE miRNA (RU vs IL; RU vs MJ); H: Positive correlated with total bacterial density in RU; I: Negatively correlated with the density of Bifidobacterium on D21 in IL; J: Positively correlated with the density of both Bifidobacterium and Lactobacillus on D42; K: Regional DE miRNA (RU vs IL; IL vs MJ), temporal DE miRNA in IL and positively correlated with the density of both Bifidobacterium and Lactobacillus on D21.

## Discussion

As an important gene expression regulatory mechanism, post-transcriptional regulation by miRNAs plays a much larger role than previously expected [34]. Although miRNAs have been widely identified from various bovine tissues [35], their roles in regulating the development and function of the GIT have not been well studied. To date, 755 miRNAs have been reported in the literature (miRBase release version 19). A recent study comparing the role of ruminant-specific miRNAs in shaping divergent mRNA expression between ruminant and non-ruminant species has revealed that at least 3.5% of genes with reduced expression in cattle can be attributed to cattle-specific miRNAs [36]. Among the detected 383 known miRNAs from calf GIT, nearly half of them were poorly conserved miRNAs, and nearly half of the poorly conserved miRNAs were bovine specific miRNAs. The number of bovine specific miRNAs is higher than the number of conserved miRNAs (Figure 2), suggesting that cattle-specific gut miRNAs may regulate gene expression that contributes to physiological and metabolic variations specific to ruminants. In addition, the detected known miRNAs and their distinct expression patterns in GIT comparing to those in other bovine tissues [37] suggests that these miRNAs may play an important role in the regional and temporal differentiation of GIT development during early life. For example, miR-143 was predominant in three gut regions and it was predicted to regulate genes involved primarily in the differentiation of connective tissue cells (Table 1). It was reported that miR-143 induced differentiation and proliferation of smooth muscle cells by targeting a group of transcription factors such as serum response factor and kruppel-like factor 4 [38], [39]. Connective tissue and smooth muscle are the major components of the gut [40], therefore the observed high expression of miR-143 throughout GIT may be associated with the rapid development and growth of the GIT during early life.

The current study also revealed a significant temporal effect on miRNA expression throughout the GIT of calves. Some of the temporal DE miRNAs revealed opposite trends between sequencing and qRT-PCR in some comparison groups, which may due to their lower number of sequencing reads that are not accurate for expression detection [41]. During early life, ruminants are considered to be monogastrics due to the bypass of milk into abomasum [42]. Less DE miRNAs from D0 to D7 and more DE miRNAs from D7 to D21 in the rumen when compared to other GIT regions may indicate slow rumen development during the first week and an accelerated development in response to a dietary change from milk alone to a diet containing solid food after 2 weeks of age. The highest number of DE miRNAs was identified during the first week after birth (D7 *vs* D0) in mid-jejunum and ileum. During this period intestinal tissues develop rapidly with exposure to many cytokines (IL-6; IL-10), growth factors (insulin-like growth factor) from maternal colostrum [43], and microbial colonization. It is not surprising that the majority of temporally DE miRNAs found in the small intestine have putative target genes involved in the development of the mucosal immune functions. For example, miR-211 has predicted targets involved in IL-6 and IL-17 cytokine signaling pathways as well as differentiation of T lymphocytes (Table 1). IL-6 stimulates Th17 T cell differentiation [44] and colonization of germ-free mice with segmented filamentous bacteria increases the number of Th17 cells [8], [45], which produce IL-17 and IL-22 to regulate the microbiota community [46]. Therefore, the detected temporal changes of miR-211 expression in mid-jejunum and ileum (Table S2) may be driven by the bacterial colonization and may be a key link between the gut microbiota and mucosal immune system development. Similarly, temporally DE miRNAs, miR-146, miR-191, miR-33, miR-7, miR-99/100, miR-486, and miR-145 may target genes that accelerate gut tissue and immune system development. The functions of miR-7 were predicted to be associated with differentiation of muscle cells (Table 1), which indicates the fast growth of muscle layers in GIT during the early life of calves. The miR-486 targets genes that mainly regulate development of fibroblast cells (Table 1), which are the most common cells in the connective tissue in the gut. Functional analysis illustrated that miR-191, miR-33, miR-99/100, and miR-145 were mainly related to differentiation of leukocytes including lymphocytes. A recent study reported that miR-99/100 targets mammalian target of rapamycin [47], and blocks the development and function of Tregs [48]. Another temporally DE miRNA, miR-146, was predicted to have multiple functions that involve in the gut connective tissue and epithelial cells development, T cell immune response and the function of dendritic cells (Table 1). The expression of miR-146 can be induced by TLR activation and it was validated to regulate TLR signaling pathways that can recognize MAMPs and play a critical role in innate immune responses [20]. The differential expression of miR-146 detected in this study indicates that gut immune system development was triggered by microbial colonization, and miR-146 may be a mediator regulating this process. This evidence supports our hypothesis that temporally DE miRNAs play a role in regulating the development of the gut mucosal immune system during early life. It is important to mention that the temporal effect in the present study is confounded by calf age/growth, gut microbial colonization, and calf diet. From D0 to D7 GIT tissues were exposed to maternal colostrum, milk, and microbial colonization, while from D14, a calf dietary supplement was introduced. Therefore, future studies that eliminate the confounding effects of diet by feeding animals only milk throughout the first 6 weeks of life, may provide a better understanding of the effect of microbial colonization on miRNAs expression in the GIT.

The DE miRNAs detected in different gut regions suggest that miRNAs may play a role in regulating the regional differentiation of the GIT. Therefore, it is important to determine if regional differences in miRNA expression reflect programmed developmental changes or differences in responses to the local microbiota. The miR-192/215 family was highly expressed in the small intestine of calves compared to the rumen during the first 6 weeks (Figure 5). The miR-192/215 family mainly targets mRNA of Runt-related transcription factor 1 (RUNX1) required for the generation of HSCs in mouse embryo, and plays a role in regulating the differentiation of hematopoietic cell lineages in adult mice [49], [50]. However, the role of RUNX1 in gut development is unclear. Functional analysis showed that mir-192/215 may be related to differentiation of leukocytes and the development of lymphoid tissues (Table 1). The small intestine contains gut-associated lymphoid tissues (GALTs) rich in leukocytes, which are not found in the rumen [51]. Higher expression levels of miR-192/215 family suggest an active modulation of leukocytes and lymphoid tissue development in mid-jejunum and ileum compared to the rumen. The expression of miR-194, which was predicted to repress induction and formation of mast cells (Table 1), was also higher in the small intestine compared to that in the rumen (Figure 5). Mast cells may disrupt the intestinal barrier during enteric nematode infection [52], and our results suggest that miR-194 may prevent the dysfunction of the mucosal barrier during the early life of calves [53]. MiR-205, which was highly expressed in the rumen (Figure 5), may regulate proliferation of cells for the development of rumen during early life. MiR-196 family was only detected in the ileum (Figure 5), and its expression varied over time (Table S2). This miRNA family regulates the proliferation of lymphatic endothelial cells (Table 1) by targeting endothelin receptor type B, insulin-like growth factor 1 and sprouty-related EVH1 domain-containing protein 1. Ileum is a major site for GALT development since it contains the continuous Peyer's patches (PPs) [54]. Thus it may not be surprising to observe high and regional-specific expression of miR-196 in the ileum during early life. Further studies are required to determine if miR-196 expression is associated with GALT, such as PPs and mesenteric lymph nodes. Investigations using the bovine fetus will also be important to further define what factors regulate miR-196 expression since GALT development is initiated during the second trimester of fetal development in the absence of the microbiome.

Previous studies revealed that colonization of GF mice with bacteria modulates host gene expression via miRNAs [22]. The exposure of newborn mammals to microbes stimulates developmental changes in the host immune system [55], and miRNAs may mediate such host microbial interaction [56]. The observed correlations between expression of miRNAs and density of total bacteria, *Lactobacillus* spp., and *Bifidobacterium* spp. suggest that miRNAs may also be involved in host-microbial cross talk through regulation of mRNA expression in response to changes in the number and composition of the microbial population. Higher numbers of miRNAs were correlated with the copy numbers of total bacteria, *Bifidobacterium* and *Lactobacillus* spp. at D21 (Figure 6), indicating that microbial population changes in response to the diet may influence the expression of miRNAs. It is known that diet can alter the composition of gut microbiota [57]. In our study, calf starter was introduced to the diet from D14 onwards. Therefore, the diet of D21 calves (milk and calf starter) was different from D7 calves (milk only). These changes in diet were associated with an altered density and composition of *Bifidobacterium* and *Lactobacillus* spp. in D21 and D42 calves(data not shown). Both *Bifidobacterium* and *Lactobacillus* spp. are beneficial commensal bacteria that stimulate host innate immune responses [58] and widely use as probiotics [59]. The expression of some miRNAs such as miR-29, miR-196 and miR-15/16 families were significantly correlated with the number of *Bifidobacterium* and *Lactobacillus* spp. in different ages of calves, revealing a potentially fundamental regulatory mechanisms by which probiotics have an effect. The main function of the miR-29 family is related to dendritic cells (DCs) maturation, the miR-196 family is involved in lymphangiogenesis, and the miR-15/16 family is involved in leukocytes differentiation. The DC maturation and leukocyte differentiation respond to probiotics treatment in the human gut [60]. Therefore, miRNAs may mediate host-microbial interaction during microbial colonization by responding to changes (density, composition or the presence) in microbiome, including *Bifidobacterium* or *Lactobacillus* spp. Our correlation analysis revealed an association between the expression of miR-129 and the total bacterial population in the rumen. Based on functional analysis, miR-129 was predicted to be involved in development of digestive system (Table 1) which suggests that miR-129 may regulate rumen development in response to the increasing bacterial population. Further studies on miRNA expression changes in relation to the host transcriptome and gut microbiome (metagenome and metatranscriptome) needs to be done to provide further evidence for the identified miRNAs playing an important role in mediating host-microbial interactions.

## Conclusions

This is the first study to profile miRNA expression throughout the bovine GIT during the immediate postnatal period of dairy calves. The present study revealed temporal and regional differences in miRNA expression. Based on the predicted targets of DE miRNAs, their functions are mainly involved in mucosal immune system development in the small intestine. Some of the abundant miRNAs, temporally DE miRNAs, regionally DE miRNAs, and miRNAs which are associated with bacterial density were predicted to have functions such as gut tissue development and immune system development through the development of GIT. Significant correlations between miRNAs and the abundance of gut bacteria suggest that miRNAs may provide a mechanism to respond to microbial colonization and regulate the development of the host mucosal immune system. The extensive small RNA sequencing dataset of bovine GIT tissues generated in the present study provides baseline data for future studies on GIT development and host-microbial interactions. Further studies to correlate miRNA expression and transcriptome of the same tissue are in progress and will provide direct evidences on how miRNA and mRNA expressions are integrated to mediate host-microbial interactions. Moreover, future studies are required to determine to what extent these changes are directly influenced by factors such as microbiota, age, and diet.

## Supporting Information

Figure S1 **Distribution of sequencing read length.**
(PDF)

Table S1 **Identification of Novel miRNAs. The sequences of novel miRNAs identified from bovine gut were listed.**
(XLSX)

Table S2 **Temporally DE miRNAs list. All temporally DE miRNAs identified in different tissues were listed.**
(XLSX)

Table S3 **Regionally DE miRNAs list. All Regionally DE miRNAs identified in different tissues were listed.**
(XLSX)

Table S4 **IPA functions of miRNAs selected for further analysis. A total of 47 miRNAs that belonged to 33 families were selected, and their predicted significant IPA function categories were listed.**
(XLSX)
