# Supplementary figures and images for: Potential Regulatory Role of MicroRNAs in the Development of Bovine Gastrointestinal Tract during Early Life
Source: PLoS One. 2014 Mar 28;9(3):e92592. doi: 10.1371/journal.pone.0092592 (PMC3969364; doi:10.1371/journal.pone.0092592)

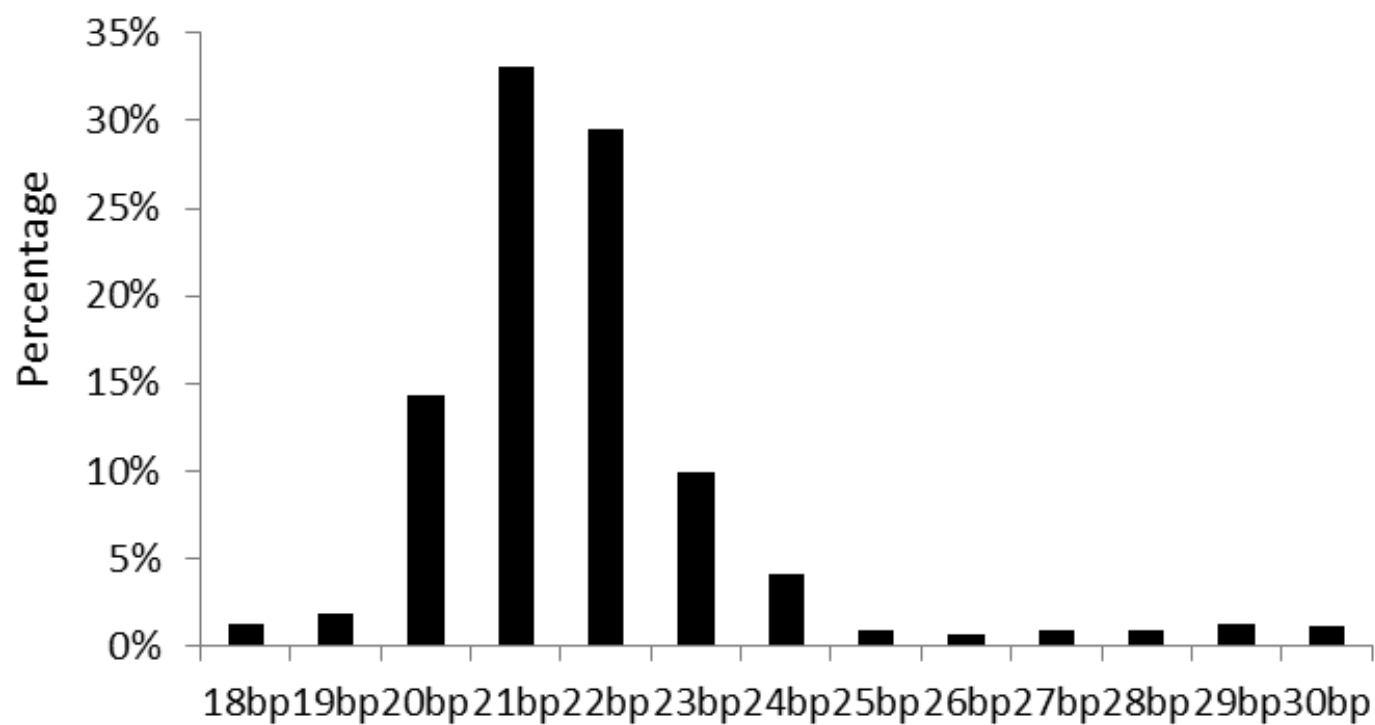

Liang et al. Figure S1

Supplement: Figure S1 — Distribution of sequencing read length. (PDF) [file pone.0092592.s001.pdf]
